# Antibiotic Expenditure by Public Healthcare Institutions in Shandong Province in China, 2012–2016

**DOI:** 10.3389/fphar.2018.01396

**Published:** 2018-12-03

**Authors:** Jia Yin, Caixia Wu, Xiaolin Wei, Qiang Sun

**Affiliations:** ^1^School of Health Care Management, Shandong University, Jinan, China; ^2^NHC Key Laboratory of Health Economics and Policy Research, Shandong University, Jinan, China; ^3^Department of Pharmacy, Shandong Medical College, Linyi, China; ^4^Dalla Lana School of Public Health, University of Toronto, Toronto, ON, Canada

**Keywords:** antibiotics, expenditure, public healthcare institutions, stewardship, zero mark-up, China

## Abstract

**Background:** Overuse of antibiotics may not only increase the burden of antimicrobial resistance, but also accelerate the growth of healthcare expenditures. China has issued a series of policies to improve antibiotic use and reduce the cost of medicine in healthcare institutions. This study aimed to evaluate the trends of antibiotic expenditure in public healthcare institutions in Shandong from 2012 to 2016 and to assess antibiotic expenditure by drug class and the level of healthcare institutions.

**Methods:** We collected data from the centralized bidding procurement (CBP) system in Shandong province between 2012 and 2016. Governmental health facilities including secondary and tertiary hospitals, and urban and rural primary healthcare centers (PHCs) procured antibiotics via this system. Antibiotics were classified according to the Anatomical Therapeutic Chemical (ATC) classification system. Antibiotic expenditure was assessed using total annual expenditure (US dollars) and expenditure per person per year (US dollars).

**Results:** The overall antibiotic expenditure was $717 million in 2016, a 56% increase compared to 2012. Parenteral antibiotics accounted for 84% of the total antibiotic expenditure in 2016. Most of the antibiotics were procured by secondary and tertiary hospitals (33 and 44%, respectively). The expenditures continuously increased in secondary hospitals, tertiary hospitals, and urban PHCs from 2012 to 2016, while antibiotic procurement decreased in urban PHCs since 2015. The third-generation cephalosporins (J01DD) were among the top five ATC classes of antibiotics in all healthcare institutions. Fluoroquinolones (J01MA) were commonly procured by tertiary hospitals, rural and urban PHCs. The expenditure on carbapenems (J01DH) raised sharply in tertiary hospitals.

**Conclusions:** The overall antibiotic expenditure kept increasing in the public healthcare institutions in Shandong. The trends of increasing expenditure began to decline in 2016, which may be associated with antibiotic stewardship initiatives. The expenditure for expensive and critical important classes of antibiotics increased, therefore it is of importance to develop policies on improving the rational use of antibiotics.

## Introduction

Globally, attention is being drawn to antimicrobial resistance (AMR). AMR has posed a serious threat not only to human health but also to economic and social development. In the absence of no effective strategy to control AMR, it is estimated that by 2050, there will be approximately 10 million deaths each year and a cumulative $100 trillion economic burden, caused by resistant infections (O'Neill, 2016). AMR is known to be associated with the irrational use of antibiotics (Goossens et al., 2005; Toska and Geitona, 2015). Improving the use of antibiotics could help reduce AMR and the subsequent costs related to it (Wang et al., 2016; Davey et al., 2017).

In China, national health expenditure sharply increased from $423 billion (based on the value of 1 US$ = 6.64 RMB in 2016) in 2012 to $698 billion in 2016 (National Health Family Planning Commission, 2017a). This increase was partly driven by the expansion of healthcare coverage and partly due to the growth of unnecessary or inappropriate health services such as the use of medicines and tests (Liang et al., 2014). A study from 47 countries found that medicines accounted for the greatest total out-of-pocket health expenditure (Alsan et al., 2015). Thus, successful control of the rise in medicine costs, is key in reducing healthcare expenditure and promoting a healthcare system reform (Shi et al., 2014). The expenditure on antibiotics was the highest of any medicine in China, however, antibiotics were commonly reported to be inappropriately used by healthcare providers (Han et al., 2013; Wang et al., 2014; Wei et al., 2017). A recent study has found that each Chinese person consumed six times more antibiotics compared to a person in the US or European countries (Zhang Q. Q. et al., 2015).

To improve the use of antibiotics and to control increasing expenditure on medicines, the Chinese government has continuously released relevant policies since 2009. In 2009, a drug centralized bidding procurement and supply chain system (hereafter short for CBP system) was established at provincial-level across China, with the purpose of supporting the essential medicine policy, regulating medicine procurement behaviors by health providers and reducing medicine costs (National Health Family Planning Commission, 2009). Public healthcare institutions were asked to negotiate and transact with pharmaceutical companies via the CBP system. Since 2013, interventions were implemented throughout China to improve antibiotic use in public hospitals (National Health Family Planning Commission, 2013). By the end of 2015, four governmental departments jointly issued a document of suggestions on controlling the irrational increase of medical expenditure in public healthcare institutions (National Health Family Planning Commission, 2015). Secondary and tertiary hospitals were required to gradually reduce their medicine cost proportion to 30%. In 2009, a zero mark-up policy was introduced to primary healthcare centers (PHCs), followed by secondary hospitals in 2012, and tertiary hospitals in 2016 (National Health Family Planning Commission, 2009; Mao and Chen, 2015). This policy is meant to prevent and eliminate revenue from prescribing.

We previously published a study regarding antibiotic consumption at the public healthcare institutions in Shandong (Yin et al., 2017). Our data showed that both oral and parenteral antibiotic consumption increased first and then declined between 2012 and 2016. The turning point appeared in 2014 for rural PHCs, and in 2015 for secondary and tertiary hospitals. Due to scarcity of available data, published literature regarding antibiotic expenditure both at national- and provincial-level, is limited. The main aim of this study was to explore trends of antibiotic expenditure at public healthcare institutions in Shandong, over a 5-year period, using antibiotic procurement data collected from Shandong CBP system. The secondary aims were to assess antibiotic expenditure of different levels of healthcare institutions as well as according to the Anatomical Therapeutic Chemical (ATC) classification system.

## Materials and Methods

### Study Setting

In 2016, Shandong had a population of 99.5 million, about 1.5 times the population of the UK (World Bank, 2016; National Bureau of Statistics of China, 2017). In 2016, the per capita GDP was $10,351 in Shandong which ranked ninth in all 31 provinces (including four independent municipalities) in China. The annual disposable income for urban and rural residents was $5,122 and $2,102, respectively (National Bureau of Statistics of China, 2017). In China, healthcare institutions are classified into tertiary hospitals, secondary hospitals and PHCs, based on the number of beds, staff numbers, types of clinical departments, infrastructures and equipment, et al. (Chinese Ministry of Health, 1989). Tertiary hospitals provide clinical, academic, and research services to the whole province or country, while secondary hospitals provide services to residents across the city or county. PHCs, including community health centers and community health stations in urban areas and township hospitals and village clinics in rural areas, are the basic units of healthcare institutions. PHCs are responsible for providing basic health services, for instance, the treatment of common diseases, vaccination, the management of non-communicable diseases and providing health education to the community. According to the China Health Statistics Yearbook there were, in total, 116 public tertiary hospitals, 347 public secondary hospitals, and 2,143 public PHCs in Shandong in 2015 (National Health Family Planning Commission, 2016).

### Data Source

Since 2011, all governmental healthcare institutions in Shandong started to procure medicines using the CBP system. The CBP system was however only fully operational toward the end of 2011. Data were thus obtained for the period 2012–2016. The CBP system records the following information: name of purchaser and date of purchase; international non-proprietary name of medicine; strength; dosage form and package; purchased quantity and unit price. Each record represented a purchase of an antibiotic by one healthcare institution. In 2016, there were a total of 2,723 public healthcare institutions in Shandong, including 116 tertiary hospitals [58 general hospitals, 20 Traditional Chinese Medicine (TCM) hospitals and 28 specialized hospitals], 368 secondary hospitals (229 general hospitals, 106 TCM hospitals, and 33 specialized hospitals), 2,152 PHCs (578 urban PHCs and 1,574 rural PHCs), and 87 other healthcare institutions (Maternity & child healthcare hospital and tuberculosis dispensary), that obtained antibiotics via the CBP system in Shandong (Table 1).

**Table 1:** Number of public healthcare institutions in Shandong Province, 2012–2016.

	**2012**	**2013**	**2014**	**2015**	**2016**
Tertiary hospital	96	96	108	116	116
General hospital	60	60	64	67	68
Hospital of TCM	18	18	20	20	20
Specialized hospitals	18	18	24	29	28
Secondary hospital	325	328	340	346	368
General hospital	220	216	216	219	229
Hospital of TCM	80	83	91	94	106
Specialized hospitals	25	29	33	33	33
PHC	2156	2156	2189	2134	2152
Urban PHC	560	559	598	537	578
Rural PHC	1596	1597	1591	1597	1574
Others (Maternity & child healthcare hospital and TB dispensary)	95	95	102	110	87

*TCM, Traditional Chinese Medicine; PHC, Primary healthcare center; TB, tuberculosis*.

### Data Analysis

All the procured antibiotics were classified and coded according to the ATC-classification system as per WHO guidelines (WHO Collaborating Centre for Drug Statistics Methodology, 2013). Antibiotics procured from the CBP system included antibacterials for systemic use (J01), antimycotics for systemic use (J02), and intestinal anti-infectives (A07A). Antibiotic expenditure was assessed using the total annual expenditure for antibiotics (US dollars) and expenditure per person per year for antibiotics (US dollars). We multiplied the purchased quantity of each procurement by unit price to get the cost for each procurement. Then, the total expenditure was calculated by adding up the cost for each procurement. The expenditure per person per year was calculated as the total annual expenditure/total population of that year. The population figures we used were based on the year-end population reported by the Statistical Year Reports of Shandong (2013–2017). We used the compound annual growth rate (CAGR) to represent the changes in the total antibiotic expenditure over time. The CAGR was calculated as (SUEnd/SUStart) ∧ (1/N) – 1, in which SUEnd is the total antibiotic expenditure for the last reported year, SUStart is the total antibiotic expenditure for the first reported year, and *N* is the number of years between the first and last year of reporting (Schellack et al., 2017).

## Results

### Antibiotic Expenditures by Formulation

**Figure 1:**
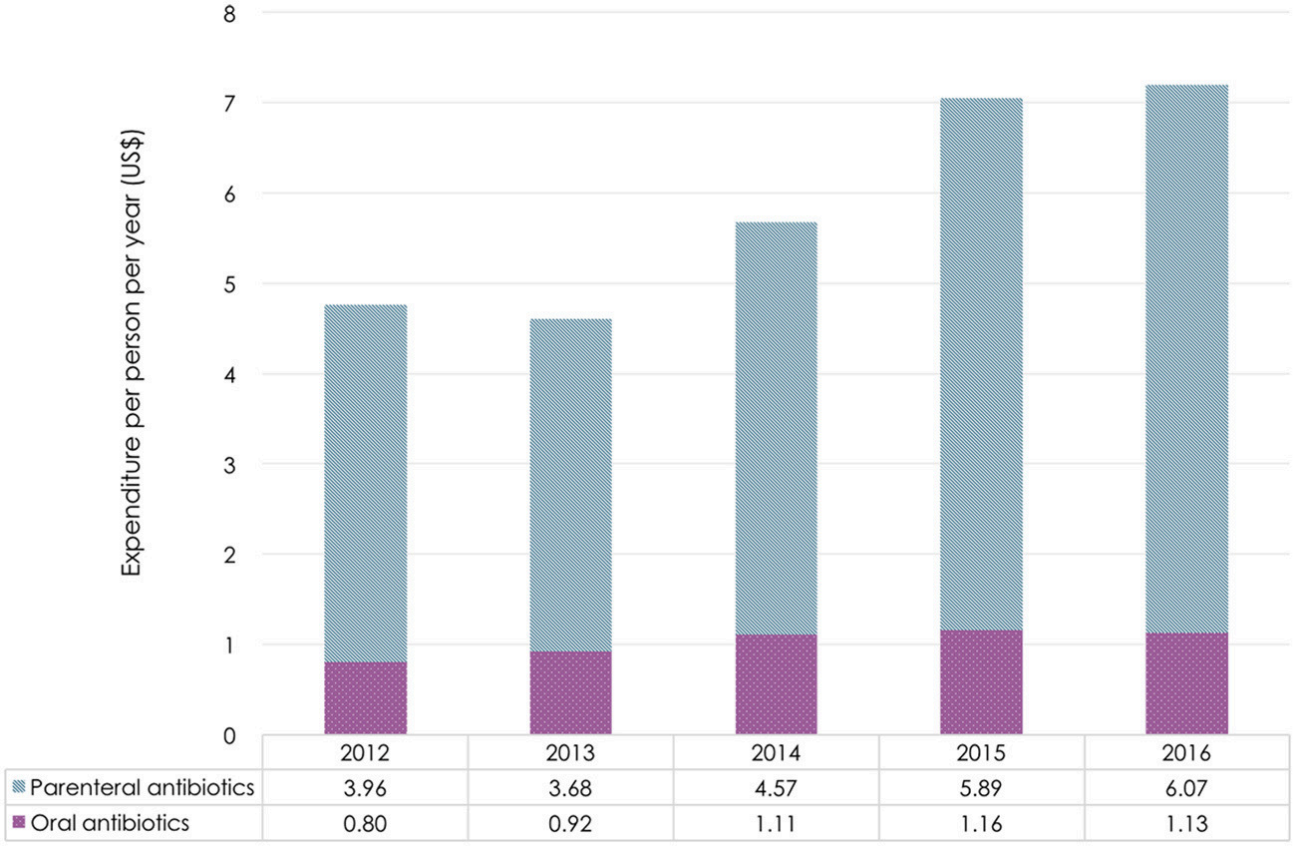
Antibiotic expenditure by dosage form in Shandong Province, 2012–2016. Based on the average exchange rate in 2016: 1 US$ = 6.64 RMB.

The overall antibiotic expenditure of public healthcare institutions in 2016 was $717 million, 56% higher than in 2012 ($460 million). From 2012 to 2016, the CAGR was 10.9% for overall antibiotic expenditure, 11.3% for parenteral antibiotic expenditure, and 9.0% for oral antibiotic expenditure. The major portion of expenditure per person per year ($6.1/$7.2, 84% in 2016) was for parenteral antibiotics. With the exception of 2013, antibiotic procurements steadily increased between 2012 and 2016. Expenditure on parenteral formulations kept increasing from 2013 to 2016. Expenditure on oral antibiotics increased from 2012 to 2014, after which it did not change much in the next 2 years (Figure 1).

### Antibiotic Expenditure by Healthcare Institution

**Figure 2:**
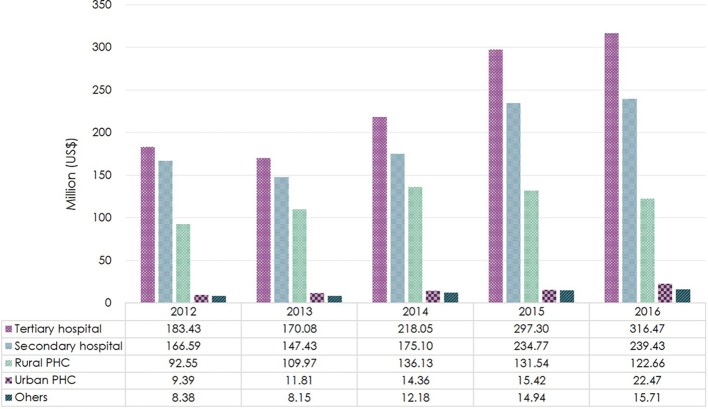
Total antibiotic expenditure by level of healthcare institution in Shandong Province, 2012–2016. Based on the average exchange rate in 2016: 1 US$ = 6.64 RMB. PHC, Primary healthcare center.

Tertiary hospitals accounted for the highest expenditures across all levels of healthcare institutions ($316 million/$717 million, 44% in 2016), followed by secondary hospitals ($239 million/$717 million, 33% in 2016). Compared to 2012, antibiotic expenditure in secondary and tertiary hospitals increased by 44 and 72% in 2016, respectively. In rural PHCs, antibiotic expenditure rose by 47% from 2012 to 2014, then declined by 10% from 2014 to 2016. Even after a 2.4-fold increase, the expenditure in urban PHCs only accounted for 4% ($22 million/$717 million) of the overall antibiotic expenditure in 2016 (Figure 2).

**Figure 3:**
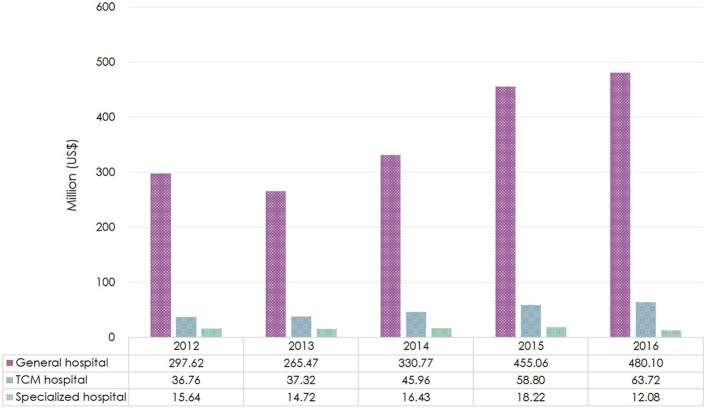
Total antibiotic expenditure by type of healthcare institution in Shandong Province, 2012–2016. Based on the average exchange rate in 2016: 1 US$ = 6.64 RMB. TCM, Traditional Chinese Medicine.

Among the secondary and tertiary hospitals, most antibiotics were procured by general hospitals ($480 million/$556 million, 86% in 2016). From 2013 to 2016, the expenditure increased yearly in both general and TCM hospitals. With a small proportion of the overall antibiotic expenditure ($12 million/$556 million, 2% in 2016), the expenditure in specialized hospitals reduced by a third in 2016 in comparison to 2015 (Figure 3).

### Antibiotic Expenditure by Class of Antibiotics

**Figure 4:**
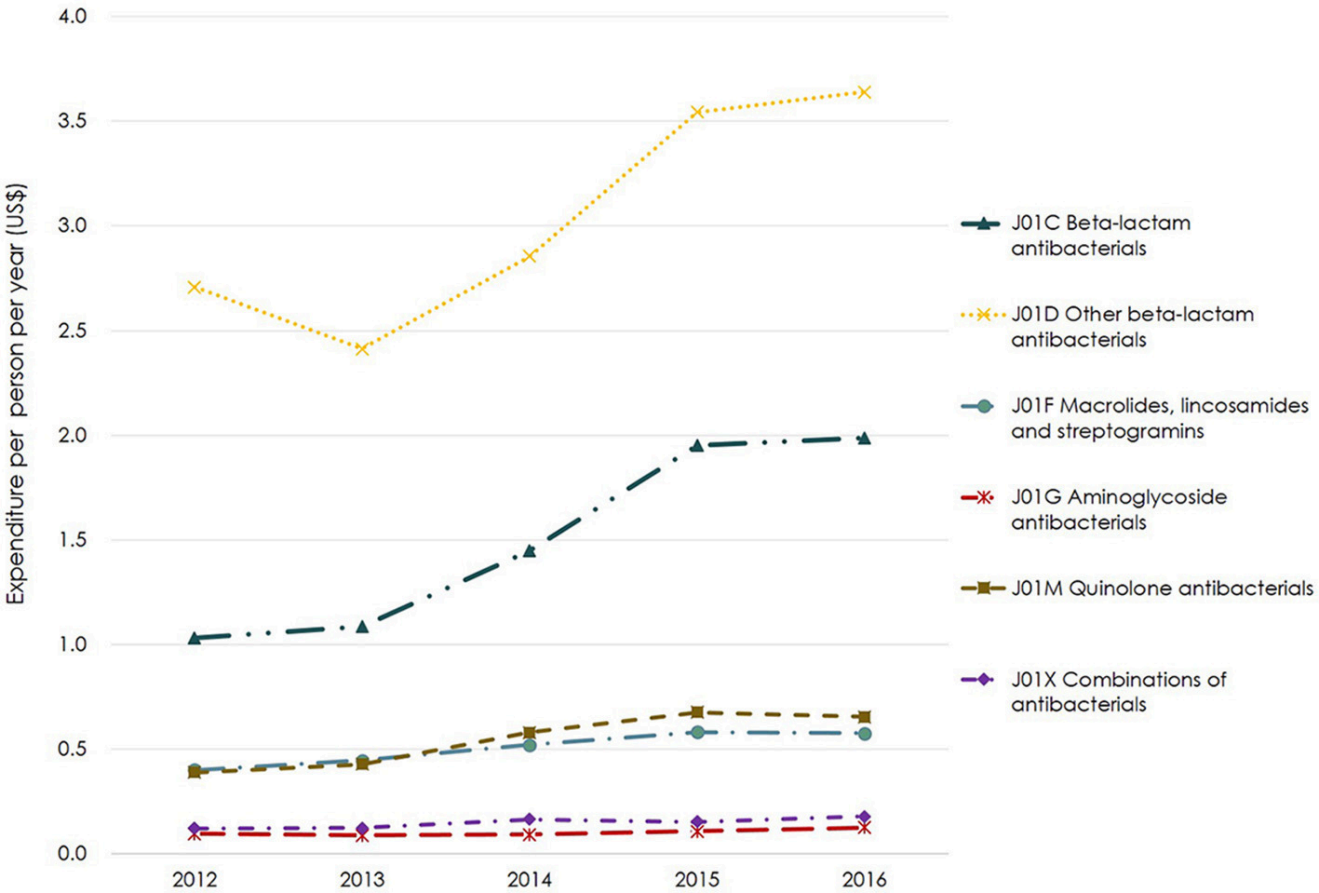
Expenditure for major classes of antibiotics based on ATC-3 in Shandong Province, 2012–2016. Based on the average exchange rate in 2016: 1 US$ = 6.64 RMB. ATC-3, The 3rd level of ATC which is classified according to pharmacological subgroup.

There were six classes of antibiotics with an expenditure of more than $0.01 per person per year, as shown in Figure 4. The expenditure for almost all classes of antibiotics increased yearly. Other beta-lactam antibacterials (J01D) was the class of antibiotics with the highest expenditure per person per year followed by beta-lactam antibacterials (J01C) and quinolones (J01M). The expenditure on other beta-lactam antibacterials (J01D) increased from $2.7 per person per year in 2012 to $3.6 per person per year in 2016. Expenditure on beta-lactam antibacterials (J01C) was the fastest-growing expenditure, which had almost doubled in 2016 compared to 2012 (Figure 4). Other classes of antibiotics did not change remarkably over the 5-year period.

### Expenditure for Different Classes of Antibiotics by Healthcare Institution

The second- and third-generation cephalosporins (J01DC and J01DD) were always among the top five classes of antibiotics by expenditure. Among the third-generation cephalosporins (J01DD), the one with the greatest expenditure was cefoperazone and beta-lactamase inhibitor (J01DD61) in tertiary hospitals, ceftizoxime (J01DD07) in secondary hospitals and ceftriaxone (J01DD04) in PHCs. Whereas, the top antibiotic by expenditure among the second-generation cephalosporins was cefotiam (J01DC07) in secondary and tertiary hospitals and cefuroxime (J01DC02) in PHCs. Expenditure on fluoroquinolones (J01MA) were among the top five in tertiary hospitals and PHCs. Since 2014, carbapenems (J01DH) which showed the fastest-growing expenditure, had replaced the first-generation cephalosporins (J01DB) to become one of the top five ATC antibiotic classes in tertiary hospitals. In secondary hospitals, combinations of penicillin (J01CR) an antibiotic class with the highest expenditure in 2016, almost tripled in expenditure over the 5-year period. Among this class, piperacillin and beta-lactamase inhibitor (J01CR05) led the expenditures in secondary and tertiary hospitals, while amoxicillin and beta-lactamase inhibitor (J01CR02) had the highest expenditure in PHCs. After 2014, the expenditure for almost all antibiotics declined in rural PHCs. On the contrary, all classes of antibiotics in urban PHCs kept increasing in expenditure during the 2012–2016 period, among which, the second-generation cephalosporins (J01DC) and combinations of penicillin (J01CR) were almost tripled in 2016 in comparison to 2012 (Table 2).

**Table 2:** Total expenditure for the top five classes of antibiotics based on ACT-4 at different levels of healthcare institutions in Shandong, 2012–2016 (million US$).

**Institutions**	**2012**		**2013**		**2014**		**2015**		**2016**	
	**ATC**	**Expenditure**	**ATC**	**Expenditure**	**ATC**	**Expenditure**	**ATC**	**Expenditure**	**ATC**	**Expenditure**
Tertiary hospital	J01DC	234.7	J01DD	233.2	J01DD	297.5	J01DD	386.2	J01DD	397.6
	J01DD	231.6	J01DC	184.1	J01DC	245.8	J01DC	268.1	J01DH	319.4
	J01MA	126.8	J01MA	147.1	J01MA	191.7	J01DH	234.6	J01DC	266.5
	J01DB	117.9	J01CR	130.1	J01CR	147.1	J01CR	234.4	J01CR	257.2
	J01CR	102.6	J01DB	105.5	J01DH	139.4	J01MA	233.6	J01MA	234.8
Secondary hospital	J01DD	211.9	J01DD	205.1	J01DD	226.8	J01CR	322.8	J01CR	353.7
	J01DC	168.6	J01DC	168.1	J01DC	223.5	J01DD	301.0	J01DD	317.5
	J01CR	139.3	J01CR	146.7	J01CR	208.5	J01DC	277.6	J01DC	255.7
	J01DF	114.2	J01DB	101.4	J01CA	133.4	J01CA	192.4	J01CA	189.3
	J01DB	104.9	J01CA	94.1	J01DB	117.9	J01DB	135.8	J01DB	142.8
Rural PHC	J01DD	110.2	J01DD	128.3	J01CR	161.9	J01CR	156.5	J01CR	146.3
	J01FA	84.9	J01FA	107.4	J01DC	137.1	J01DC	142.2	J01DC	131.8
	J01DC	79.1	J01DC	95.9	J01DD	109.4	J01DD	103.1	J01DD	95.5
	J01CR	79.0	J01CR	93.6	J01FA	109.0	J01FF	92.6	J01FF	80.7
	J01CE	58.7	J01DB	69.8	J01MA	78.4	J01FA	83.6	J01FA	77.8
Urban PHC	J01DD	10.1	J01DD	12.2	J01DC	17.0	J01DC	19.0	J01DC	26.3
	J01DC	9.4	J01FA	12.1	J01CR	13.5	J01CR	15.7	J01CR	24.1
	J01FA	9.0	J01DC	11.7	J01FA	13.0	J01MA	12.2	J01FA	17.5
	J01CR	7.2	J01CR	10.2	J01MA	11.2	J01DD	12.0	J01DD	16.0
	J01DB	5.5	J01CE	5.7	J01DD	10.7	J01FA	11.7	J01MA	16.0

*Based on the average exchange rate in 2016, 1 US$, 6.64 RMB. ATC-4, the 4th level of ATC which is classified according to chemical subgroup; PHC, Primary healthcare center. J01CA, Penicillin with an extended spectrum; J01CE, Beta-lactamase sensitive penicillin; J01CR, Combinations of penicillin, incl. beta-lactamase inhibitors, J01DB, First-generation cephalosporins, J01DC, Second-generation cephalosporins; J01DD, Third-generation cephalosporins; J01DF, Monobactams; J01DH, Carbapenems, J01FA, Macrolides; J01FF, Lincosamides; J01MA, Fluoroquinolones*.

## Discussion

There was an increasing trend in antibiotic expenditure in Shandong between 2012 and 2016, which could be largely attributed to the increased procurement of parenteral antibiotics. Most antibiotic expenditure occurred in secondary and tertiary hospitals. In secondary hospitals, tertiary hospitals and urban PHCs, which are often located in urban areas, expenditure continued to increase during the study period. However, in rural PHCs, we found that expenditure began to decrease in 2015. The top ATC antibiotic class was other beta-lactam antibacterials (J01D), followed by beta-lactam antibacterials (J01C) and quinolones (J01M). The third-generation cephalosporins (J01DD) class was among the top five ATC antibiotic classes in all public healthcare institutions from 2012 to 2016. Fluoroquinolones (J01MA) have been in the top five ATC antibiotic classes in tertiary hospitals and PHCs. The rank of carbapenems (J01DH) class was on the rise in tertiary hospitals.

Antibiotic stewardship initiatives have helped to decrease both antibiotic consumption and expenditure in the United States, based on the national data reported during 2010 and 2015 (Van Boeckel et al., 2014; Suda et al., 2018). In China, the consumption of antibiotics still stably increased yearly (Van Boeckel et al., 2014; Wushouer et al., 2017). In 2015, the antibiotic consumption in Shandong was 5–8 DDD per 1,000 inhabitants per day, which represented a median level of antibiotic consumption in China (Wushouer et al., 2017). Our previous study found that the consumption of both oral and parenteral antibiotics had decreased in Shandong since 2014, however, this study showed an upward trend in expenditure during the same period (Yin et al., 2017). This increase was mainly attributed to the growth of expenditure on parenteral formulations. It indicated that although the consumption of oral or parenteral antibiotics with lower unit prices had declined, the consumption of expensive parenteral antibiotics kept rising instead.

In accordance with global evidence, about 80% of all antibiotics were consumed in PHCs (Center for Disease Dynamics Economics Policy, 2015). The 2013 intervention which required public healthcare institutions to prescribe no more than two antibiotics in one outpatient visit and the 2014 policy of improving antibiotic use in PHCs have contributed to a reduction in antibiotic consumption in rural PHCs where antibiotics were more likely to be irrationally used (National Health Family Planning Commission, 2013, 2014; Yin et al., 2017). As rural PHCs often have a limited selection of antibiotics available, a decline in consumption directly caused the reduction of antibiotic expenditure in rural PHCs.

Regarding the total medicine expenditure, inconsistent with the findings in the United States where PHCs spent the most for antibiotics, secondary and tertiary hospitals paid more for antibiotics than PHCs in Shandong (Suda et al., 2018). This is also contrary to our earlier study regarding antibiotic consumption, which showed that most antibiotics were consumed in rural PHCs in Shandong (Yin et al., 2017). The potential reason was that secondary and tertiary hospitals were more likely to prescribe parenteral antibiotics than the PHCs in China. In late 2015 and mid-2016, the Chinese government had issued policies to control the medicine cost proportion and zero mark-up in public hospitals. These policies may help to reduce the increasing rate of antibiotic expenditure in secondary and tertiary hospitals, as we have observed in this study. Previous studies have also confirmed that the effect of the zero mark-up policy, on decreasing drug expenditure in secondary hospitals and rural PHCs in China (Zhang et al., 2014; Zhang H. et al., 2015).

Cephalosporins and quinolones were among the top used ATC classes of antibiotics in many countries including China, while at the same time increased rates of bacteria resistance to these antibiotics were reported (Liew et al., 2011; Van Boeckel et al., 2014; Kourlaba et al., 2015). According to the WHO Critically Important Antimicrobials (CIA) list, third-generation cephalosporins and quinolones were among the highest priority classes of antibiotics and should be used prudently (World Health Organization, 2017). Whereas, both the third-generation cephalosporins and fluoroquinolones had high expenditures in our study. Carbapenems, which were recognized as the last line of defense for bacterial infections, were classified as special antibiotics and should not be used without consultation with infection control experts in China (National Health Family Planning Commission, 2017b). However, an uptrend of expenditure for carbapenems as well as consumption was observed in tertiary hospitals in China (Wushouer et al., 2017). These findings suggest that the current policies are obviously effective in controlling overall consumption and expenditure, but have very little impact on improving the rational use of antibiotics.

This study reported the trends of antibiotic expenditure in a province, covering about one-tenth of the total national population, based on the CBP system. It filled a research gap where previous studies only focused on antibiotic cost data collected from prescriptions. However, several limitations should be acknowledged. Firstly, we cannot calculate expenditure per user as no patient-level data were recorded in the CBP system. Further studies in this field, using data based on a patient-level, such as data from the health insurance system, would be greatly beneficial. Secondly, we did not consider antibiotic expenditure that may have occurred in private pharmacies. Thus, the total antibiotic expenditure at provincial-level may be underestimated. Thirdly, the trends of antibiotic expenditure in public healthcare institutions were evaluated based on years. We suggest that future studies evaluate monthly trends to shed additional light on diagnostic demands within years. Fourth, the increasing number of secondary and tertiary hospitals may impact on the changes in expenditure. Finally, the policies of zero mark-up and medicine cost proportion controlling were issued in recent years. Future studies which evaluate the long-term effect of these policies on antibiotic expenditure are therefore recommended.

## Conclusion

The overall antibiotic expenditure increased over time in Shandong, China. However, the increase rate of expenditure began to decline in 2016, possibly related to the implementation of antibiotic stewardship initiatives. The expenditure for expensive and critically important classes of antibiotics continued to increase in public healthcare institutions, especially in tertiary hospitals. In addition to controlling the overall expenditure, policies should be developed to improve the rational use of antibiotics in public healthcare institutions.

## Author Contributions

JY and QS designed the study. CW helped to collect and interpret the data. JY analyzed the data and wrote the first draft of the manuscript. QS and XW provided critical comments and revised the manuscript. All authors reviewed the manuscript.

### Conflict of Interest Statement

The authors declare that the research was conducted in the absence of any commercial or financial relationships that could be construed as a potential conflict of interest.
